# Supplementary material for: Loneliness and diurnal cortisol levels during COVID-19 lockdown: the roles of living situation, relationship status and relationship quality
Source: Sci Rep. 2022 Sep 5;12:15076. doi: 10.1038/s41598-022-19224-2 (PMC9443629; doi:10.1038/s41598-022-19224-2)
Supplement: Supplementary file 1 — Supplementary Information. [file 41598_2022_19224_MOESM1_ESM.docx]

# **Appendix**

## **Appendix A - Formulas of the hypotheses 4 (1) and 5 (2).**

1. **Level 1:** lnCort_ij_ = ß_oi_ + ß_1_ *C_lonely_ij_* + ß_2_ *eat_ij_* + ß_3_ *drink_ij_* + ß_4_ *alcohol_ij_* + ß_5_ *caffeine_ij_* + ß_6_ *physical activity_ij_* + ß_7_ *time_rise_ij_* + ß_8_ *time_fall_ij_* + ß_9_ *day_ij_* + ε_ij_

**Level 2:** ß_0j_ = γ_00_ + γ_10_ *GC_lonely* + υ_0i_

1. **Level 1:** lnCort_ij_ = ß_oi_ + ß_1_ *C_lonely_ij_* + ß_2_ *C_lonely_ij_*relationship._i._* + ß_3_ *C_lonely*living_.j_* + ß_4_ *eat_ij_* + ß_5_ *drink_ij_* + ß_6_ *alcoho*l_ij_ + ß_7_ *caffeine**_ij_* + ß_8_ *physicalactivity_ij_* + ß_9_ *time_rise_ij_* + ß_10_ *time_fall_ij_* + ß_11_ *day_ij_* + ε_ij_

**Level 2:** ß_0j_ = γ_00_ + γ_10_ *GC_lonely_.j_* + γ_11_ *age_.j_* + γ_12_ *sex._j_* + γ_13_ *bmi_.j_* υ_0j_

Where i denotes the measurement nested in person j, vector *C_lonely* captures person-mean-centered momentary loneliness levels. *GC_lonely* captures grand-mean-centered loneliness varying on the person level (level 2), and *relationship* (0 = Single, 1 = In a relationship) and *living* (0 = Alone, 1 = With others) characteristics also varying on level 2. The vectors *C_lonely_ij_*relationship_i_* and *C_lonely*living_.j_* represent cross-level interactions, with *relationship_.j_* and *living_.j_* being level 2 predictors. Finally, ε_ij_ denotes individual variations, whereas υ_0i_ represents differences between each person’s mean from the global mean.

Pseudo R² of the significant predictors was calculated as follows: (σ²_0_ - σ²_1_)_/_ σ²_0_

Where σ²_0_ denotes the amount of variance explained before including the predictor, and σ²_1_ denotes the amount of variance explained after including the predictor.

## **Appendix B – Supplementary tables with results of the multilevel models**

**Table 1**

*Results of the reduced multilevel models with loneliness, relationship status and living situation as predictors and cortisol levels (ln-transformed) as outcome.*

| **Effects** | **Fixed Slopes Model** | **Random Intercept and Slopes Model** |
| --- | --- | --- |
| Fixed effects |  |  |
| Intercept | 2.889** (.097) | 2.896** (.096) |
| Within-person effect (L1)  Loneliness | 0.002^#^ (.001) | 0.002 (.002) |
| Between-person effects (L2)  Loneliness  Relationship status  Living situation | 0.0003 (.001)  -0.079* (.035)  -0.019 (.039) | 0.0004 (.001)  -0.078* (.035)  -0.018 (.039) |
| Cross-Level interaction (L1xL2)  Relationship status*Loneliness  Living situation*Loneliness | -0.004* (.001)  0.001 (.001) | -0.004* (.002)  0.001 (.002) |
| Covariates  Age  Sex  Day  Body Mass Index (BMI)  Eating (yes/no)  Drinking (yes/no)  Alcohol (yes/no)  Caffeine (yes/no)  Physical activity (yes/no)  Time-rise^a^  Time-fall^b^ | 0.001 (.001)  0.031 (.033)  -0.025 (.017)  -0.01* (.003)  0.049 (.031)  -0.124** (.034)  -0.048 (.038)  0.054* (.023)  -0.004 (.022)  -0.172 (.252)  -0.501** (.013) | 0.001 (.001)  0.036 (.033)  -0.026 (.017)  -0.011* (.003)  0.054^#^ (.031)  -0.130** (.034)  -0.052 (.038)  0.053* (.023)  -0.003 (.022)  -0.144 (.249)  -0.501** (.013) |
| Random effects (standard deviation)  Intercept  Loneliness  Residual | .189  -  .340 | .190  .004  .336 |

*Note.* Table depicts unstandardized coefficients (standard errors in parentheses). Number of observations = 1722; Number of participants = 225. a -2 = time-point 1; -1 = time-point 2; 0 = time-points 3-6 b 0 = time-point 1 = time-point 4; 2 = time-point 5; 3 = time-point 6. ^#^ *p* < .1; **p* < .05; ***p* < .001.

**Table 2**

*Fit indices of the multilevel model.*

| Model | df | AIC | BIC | LL | L Ratio | *p* |
| --- | --- | --- | --- | --- | --- | --- |
| 1 | 19 | 1591.11 | 1694.5 | -776.55 |  |  |
| 2 | 21 | 1587.587 | 1701.855 | -772.8 | 7.52 | .02 |

*Notes.* Model 1 = Random intercept-only model; model 2 = Random intercepts and slopes-model with level 1 - loneliness set as random predictor; df = degrees of freedom; AIC = Akaike’s information criterion (goodness of fit index); BIC = Schwarz’s Bayesian criterion (goodness of fit index); LL = Log likelihood, L Ratio = Likelihood ratio; p = p-value.

**Table 3**

*Results of the full multilevel models with loneliness, relationship status and living situation as predictors and cortisol levels (ln-transformed) as outcome.*

| **Effects** | **Fixed Slopes Model** | **Random Intercept and Slopes Model** |
| --- | --- | --- |
| Fixed effects |  |  |
| Intercept | 2.966** (.150) | 2.983** (.149) |
| Within-person effect (L1)  Loneliness | 0.004 (.002) | 0.002 (.003) |
| Between-person effects (L2)  Loneliness  Relationship status  Living situation | 0.0003 (.001)  -0.121* (.045)  -0.070 (.052) | 0.0004 (.001)  -0.121* (.045)  -0.076 (.051) |
| Cross-Level interaction (L1xL2)  Relationship status*Loneliness  Living situation*Loneliness | -0.006* (.002)  0.001 (.002) | -0.008* (.003)  0.004 (.003) |
| Covariates  Age  Sex  Day  Body Mass Index (BMI)  Eating (yes/no)  Drinking (yes/no)  Alcohol (yes/no)  Caffeine (yes/no)  Cigarettes (yes/no)  Physical activity (yes/no)  Time-rise^a^  Time-fall^b^  Sleep duration  Sleep quality  Problem falling asleep  Sleeping pills  Forced awake  Brushing teeth (yes/no) | 0.001 (.002)  0.031 (.041)  -0.053^#^ (.028)  -0.011* (.004)  0.009 (.046)  -0.08 (.051)  -0.039 (.055)  0.052 (.034)  0.127* (.064)  -0.016 (.033)  -0.081 (.273)  -0.489** (.018)  -0.001 (.003)  -0.000 (.001)  0.056 (.045)  -0.017 (.083)  -0.014 (.035)  0.025 (.029) | 0.001 (.002)  -0.028 (.041)  -0.059* (.027)  -0.011* (.004)  0.018 (.046)  -0.09^#^ (.050)  -0.042 (.055)  0.047 (.034)  0.125* (.063)  -0.003 (.022)  -0.043 (.269)  -0.49** (.018)  -0.000 (.003)  -0.000 (.001)  0.059 (.045)  -0.026 (.082)  -0.018 (.034)  0.022 (.029) |
| Random effects (standard deviation)  Intercept  Loneliness  Residual | .177  -  .353 | .177  .005  .346 |

*Note.* Table depicts unstandardized coefficients (standard errors in parentheses). Number of observations = 856; Number of participants = 157. a -2 = time-point 1; -1 = time-point 2; 0 = time-points 3-6 b 0 = time-point 1 = time-point 4; 2 = time-point 5; 3 = time-point 6. ^#^ *p* < .1; **p* < .05; ***p* < .001.

**Table 4**

*Fit indices of the multilevel model.*

| Model | df | AIC | BIC | LL | L Ratio | *p* |
| --- | --- | --- | --- | --- | --- | --- |
| 1 | 27 | 974.4111 | 1101.922 | -460.2055 |  |  |
| 2 | 29 | 970.5101 | 1107.466 | -456.2551 | 7.901 | .019 |

*Notes.* Model 1 = Random intercept-only model; model 2 = Random intercepts and slopes-model with level 1 - loneliness set as random predictor; df = degrees of freedom; AIC = Akaike’s information criterion (goodness of fit index); BIC = Schwarz’s Bayesian criterion (goodness of fit index); LL = Log likelihood, L Ratio = Likelihood ratio; p = p-value.
